# Preparation of cellular samples using graphene cover and air-plasma treatment for time-of-flight secondary ion mass spectrometry imaging†

**DOI:** 10.1039/c9ra05205d

**Published:** 2019-09-09

**Authors:** Heejin Lim, Sun Young Lee, Dae Won Moon, Jae Young Kim

**Affiliations:** Department of New Biology, Daegu Gyeongbuk Institute of Science and Technology (DGIST) 333 Techno Jungang Daero, Hyeonpung-Eup, Dalseong-Gun Daegu 42988 Republic of Korea dwmoon@dgist.ac.kr jyk@dgist.ac.kr; Division of Technology Business, National Institute for Nanomaterials Technology (NINT), Pohang University of Science and Technology (POSTECH) 77 Cheongam-Ro, Nam-Gu Pohang Gyeongbuk 37673 Republic of Korea; Department of Robotics Engineering, Daegu Gyeongbuk Institute of Science and Technology (DGIST) 333 Techno Jungang Daero, Hyeonpung-Eup, Dalseong-Gun Daegu 42988 Republic of Korea

## Abstract

We report on sample preparation methods based on plasma treatment for an improvement of multiple molecular ion images of cellular membranes in the ToF-SIMS method. The air-plasma treatment of fixed cellular samples efficiently removed the organic residues of any solutions used during sample preparation and improved the quality of ToF-SIMS images due to the resulting clean surface. We also studied cell preparation methods that combine single-layer graphene covering with air-plasma treatment to achieve a synergistic effect that eliminates background spectra by organic impurities while minimizing morphological cell deformation in a vacuum environmental analysis. When the cellular sample on the glass substrate is completely covered with the single-layer graphene, the cells trapped between the graphene and the substrate can effectively reduce morphological deformation by slow-dehydration. After slow-dehydration of cells is completed inside the graphene-cover, the covered graphene layer can be peeled off by air-plasma treatment, and the unwanted organic residues on the surface of cells and substrate can also be removed by plasma cleaning, thereby much improving ion imaging of cells with the ToF-SIMS method. It is confirmed that the cell samples in which the graphene-cover was removed by air-plasma treatment maintained their morphology well in comparison with the rapid air-dried cells in atomic force microscopy (AFM) and ToF-SIMS images.

## Abstract 2

Cell preparation methods that combine a single-layer graphene cover with air-plasma treatment for improvement of ToF-SIMS imaging.

## Graphical abstract

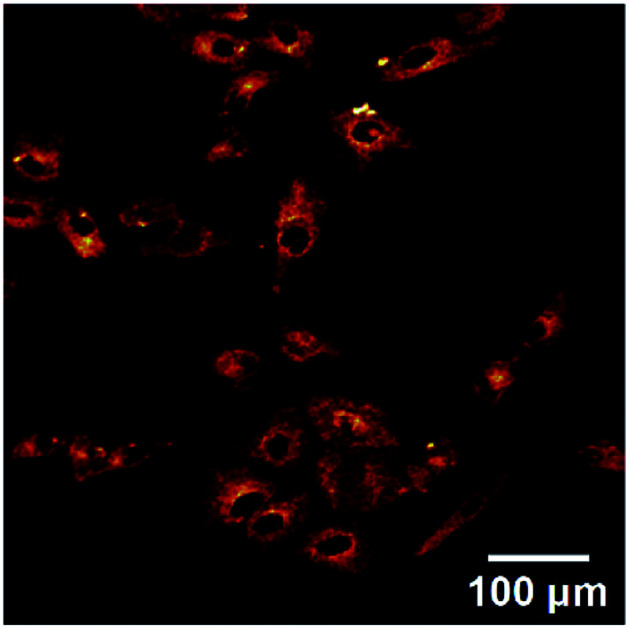

## Introduction

Time-of-flight secondary ion mass spectrometry (ToF-SIMS) is a promising mass spectrometry (MS) imaging technique with a surface specificity and submicron spatial resolution using accelerated ion beams in a vacuum environment^1,2^ and can image multiple unlabelled lipids, metabolites, and small molecules of cellular membranes in parallel.^3,4^ Since ToF-SIMS operates under an ultrahigh vacuum environment, biological samples such as cells and tissues should be properly prepared to be compatible with vacuum conditions, such as by chemical fixation followed by dehydration, freeze drying, or frozen hydration method.^5–8^ Compared to chemical fixation, frozen hydrated sample preparation has been more recognized for preserving cell morphology and native distribution of molecules including diffusible ions, as well as for enhancing molecular ion intensities for phospholipids for ToF-SIMS imaging of hydrated cells.^9,10^ Despite the aforementioned advantages, a frozen hydration method requires laborious, painstaking procedures for reproducible results, as well as special equipment like a liquid nitrogen cooled stage to keep samples frozen during the analysis. This complicated sample preparation method was unattractive to researchers in the life sciences, who are more familiar with relatively facile chemical fixed sample preparation, which has been well established for electron microscopy.

Chemical fixation generally involves crosslinking the proteins with formaldehyde or glutaraldehyde and then air-drying the samples; If necessary, samples can be post-fixed by crosslinking the lipids with osmium tetroxide before drying. Prior studies showed that chemical fixation also can maintain the fine cell-surface structure and spatial integrity of lipids in cell membranes.^8,11^ One of the common concerns in preparing cells and tissues for ToF-SIMS imaging is that extracellular medium containing biological salts such as Na^+^, K^+^, and Cl^−^ should be thoroughly removed. Otherwise, the salts contribute to isobaric interferences and reduction of organic secondary ion yields, entailing an adverse effect on the analysis and imaging of the membrane lipids.^12^ Various rinsing methods, which also should not damage samples, have been suggested to remove biological salts from the sample.^12,13^ Another main concern is that natively hydrated biological samples can be easily deformed or cracked while drying the samples.^8,14^ Although critical point drying or alcohol dehydration is an established method of dehydrating delicate biological samples while avoiding surface tension effects due to liquid water vaporization,^5,6^ the usage of alcohol was determined to lead to a significant loss of cell membrane lipids.^8^

Here, we report on facile sample preparation method using graphene and air-plasma treatment for ToF-SIMS imaging of intact cellular membranes with improved quality of multiple molecular ion images at a submicron lateral resolution. Graphene,^15^ a gas-impermeable carbon honeycomb mesh, has recently enabled electron microscopy imaging of hydrated cells under vacuum environment.^16,17^ Our protocol basically starts with chemical fixation of cells and shuns rapid air-drying by coating wet cells on a culturing glass with graphene, which helps maintain the cell morphology. The three-dimensional (3D) structure of graphene-covered cells was better preserved than that of air-dried cells, as determined by atomic force microscopy (AFM). Since ToF-SIMS is a surface-sensitive analysis that only allows analysis of the upper one to two monolayers of the sample due to the escape depth of secondary ions,^18^ even trivial contaminations on the surface can cause serious adverse effects on ToF-SIMS analysis. Graphene-covered cells were treated by air-plasma in order to not only gently remove the graphene cover, but also to remove impurities on the surface, thereby exposing clean cellular membranes to the surface.

We found that air-plasma treatment efficiently removed residual impurities of any solutions used during sample preparation and that the resulting clean surface facilitated the improved quality of ToF-SIMS images. Besides, graphene removed cells by air-plasma treatment also better maintained the morphology compared to air-dried cells as highlighted in ToF-SIMS images. We also examined graphene-covered cells before and after air-plasma treatment with different plasma-treated periods using helium ion microscopy (HIM). As a result, one minute of air-plasma treatment seems optimal to completely remove graphene without cell damage and simultaneously enhance ToF-SIMS imaging of cellular membranes by plasma cleaning.

## Materials and methods

### Time-of-flight secondary ion mass spectrometry (ToF-SIMS) imaging

ToF-SIMS analysis was conducted on a ToF-SIMS 5-100 instrument (ION-TOF, Münster, Germany) using a pulsed 30 keV Bi_3_^+^ primary ion beam in delayed extraction mode for positive and negative ion ToF-SIMS images over a 500 × 500 μm^2^ or 200 × 200 μm^2^ area with 256 × 256 pixels. Internal mass calibration for ToF-SIMS spectra was performed using the peaks of CH_3_^+^, Na^+^, C_2_H_3_^+^, C_3_H_5_^+^ and C_4_H_7_^+^ for positive ion mode and C^−^, C_2_^−^, C_3_^−^, and C_4_^−^ for negative ion mode before further analysis.

### Cell culture

A549 human lung carcinoma cells (ATCC, CCL-185) were plated on 12 mm-diameter cover glasses at 37 °C, 5% CO_2_ in an incubator and grown overnight in culture medium [Roswell Park Memorial Institute Medium (RPMI-1640) containing l-glutamine supplemented with 10% (v/v) fetal bovine serum (FBS), 50 μg per ml penicillin–streptomycin and 100 μg per ml Neomycin, all purchased from Gibco, except Neomycin from Sigma Aldrich].

### Chemical fixation and air-plasma treatment

For chemical fixation with formaldehyde, cells were washed for 5 min three times with phosphate buffered saline (Gibco, PBS) and fixed in 10% formalin solution (Sigma Aldrich) for 10 min at room temperature (RT). After fixation, the fixed cells were washed with PBS and with distilled water (DW) each three times, and then air-dried. The fixed cells were treated by air-plasma using a plasma chamber (CUTE, Femto Science Inc., South Korea) at 1.1–1.3 Torr, 50 kHz, 100 W, and 70 sccm of air for 5 min.

### Graphene transfer on cells and air-plasma treatment

Cells fixed with 10% formalin solution were used for graphene-covered cells. The entire process for sample preparation was performed as shown in Fig. S1.† CVD graphene grown on Cu foil was purchased from Graphene Platform, Japan. The backside graphene of Cu foil was etched away by O_2_ plasma reaction ion etching (RIE) and then the Cu foil was etched by 0.1 M ammonium persulfate solution (DAEJUNG, Korea) for 4 h. The graphene was rinsed in DW several times. Fixed cells stored in PBS were rinsed three times in DW, and then graphene was transferred onto the cells by scooping the culturing glass up from under graphene floating on the surface of DW and air-dried. Graphene-covered cells were treated by air-plasma using a plasma chamber (CUTE, Femto Science Inc., South Korea) at 1.1–1.3 Torr, 50 kHz, 100 W, and 70 sccm of air for 1, 3, and 5 min.

### Cellular sample characterization

For HIM imaging, HIM measurements were performed on an Orion NanoFab instrument (Carl Zeiss, USA) at 30 keV of beam energy and 0.3–0.9 pA of probe current. Electron flood gun was used to compensate charging effects for biological samples without graphene or metal coating. For Raman spectroscopy analysis, graphene transferred on cells and the same region treated by air-plasma afterward were analyzed (Nicolet Almega XR, Thermo Scientific, USA) using a 532 nm laser source. For atomic force microscopy (AFM), topographical images of air-dried cells, graphene-covered cells, and graphene-removed cells over 90 × 90 μm^2^ with 256 × 256 pixels were acquired by large sample atomic force microscope (XE-150, Park Systems, Korea) at 0.3 Hz scan rate.

## Results and discussion

### Synergy between graphene preserving cell morphology and air-plasma treatment cleaning cell surface enhances ToF-SIMS imaging

**Fig. 1:**
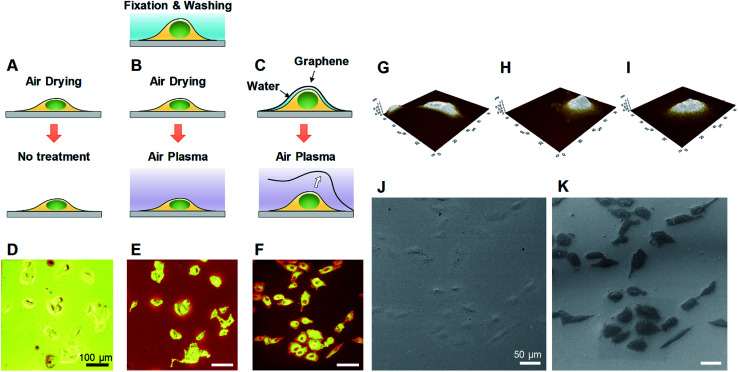
Cellular sample preparations for ToF-SIMS imaging and their characterizations. (A–C) Schematics of three different methods for preparing A549 cells after chemically fixed and water-rinsed. (D–F) ToF-SIMS images of total positive ions for A549 cells prepared by each method: drying in air (A and D), air-plasma treatment after drying (B and E), and a combination of graphene transfer, air drying, and air-plasma treatment eventually removing graphene (C and F). (G–I) AFM images for air-dried cells (G), graphene covered cells (H), and graphene removed cells (I) show that graphene enables the preservation of cellular morphology. (J and K) HIM images of graphene covered cells before (J) and after 5 min air-plasma treatment (K) reveal that graphene was completely removed by air plasma. The HIM images clearly correspond to one of the ToF-SIMS (F) for graphene-removed cells. Scale bar, 100 μm (D–F), 50 μm (J and K).

We developed a facile method of preparing well-preserved biological samples using single-layer graphene and air-plasma treatment for improvement of ToF-SIMS imaging. Illustrative schematics (Fig. 1A–C) represent three distinctive procedures of cell preparations based on chemical fixation: conventional method (water rinsing and air-drying of fixed cells), conventional method with air-plasma treatment after drying, and our proposed method, where fixed cells are covered with graphene and air-dried. Once drying is completed, air-plasma treatment is applied to the cells for the latter two procedures. Cells prepared by our protocol using both graphene and air-plasma treatment are called graphene-removed cells in this paper for convenience. The morphological distinction between air-dried cells (Fig. 1D and E) and graphene removed cells (Fig. 1F) clearly manifested as shown in total positive ions ToF-SIMS images. The topographic images of AFM reveal that cells prepared without graphene were subjected to damage while drying in air and ended up shrunk in size (Fig. 1G), whereas the cellular volume and morphology were well maintained in cells covered with graphene (Fig. 1H) and even after graphene was removed by air-plasma treatment with minor shrinkage (Fig. 1I). HIM with surface sensitivity and sub-nanometer resolution^19–21^ is useful to examine the quality of graphene transfer on cells and morphology of biological samples without metal coating; the HIM images confirmed that the graphene was completely removed from the cells by air-plasma treatment without noticeable distortion (Fig. 1J and K).

**Fig. 2:**
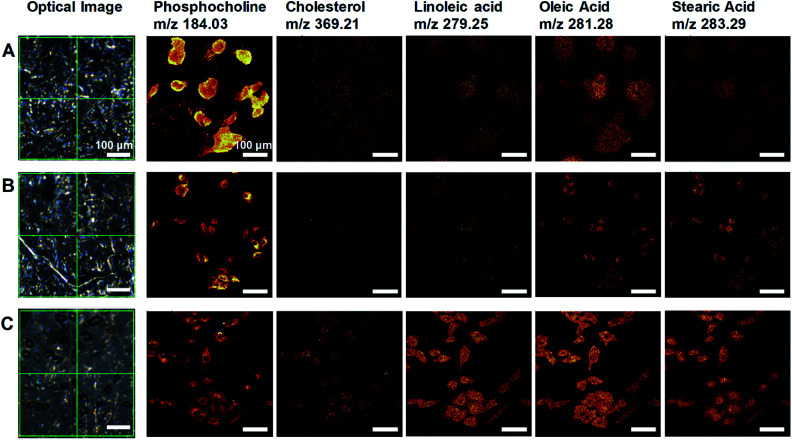
ToF-SIMS lipid imaging of A549 cell membranes prepared by three different methods. (A–C) Optical images and ToF-SIMS images of (left to right) phosphocholine, cholesterol, linoleic acid, oleic acid, and stearic acid for air-dried cells before (A) and after air-plasma treatment (B), and graphene removed cells (C). Scale bar, 100 μm (A–C).

High-quality ToF-SIMS images of graphene removed cells with submicron resolution, high contrast, and reduced background signals represented cell morphology well to match one as in HIM images of the cells. Furthermore, ToF-SIMS provides abundant information on molecular maps, particularly lipid distribution in cell membranes. The representative ToF-SIMS images (Fig. 2) for A549 cells prepared by the three different methods show the distributions of phosphocholine at C_5_H_15_NPO_4_^+^ (*m*/*z* = 184.03), cholesterol at C_27_H_45_^+^ (*m*/*z* = 369.21), and various fatty acids such as linoleic acid at C_18_H_31_O_2_^−^ (*m*/*z* = 279.25), oleic acid at C_18_H_33_O_2_^−^ (*m*/*z* = 281.28), and stearic acid at C_18_H_35_O_2_^−^ (*m*/*z* = 283.29). To examine the effects of air-plasma on ToF-SIMS imaging of cells, ToF-SIMS images of the cells before and after 5 min air-plasma treatment for air-dried fixed A549 cells were compared. Cellular samples can be easily contaminated by any substances, even washing solutions, through the preparation procedure in the atmosphere, which contributes to unwanted background mass spectra without air-plasma treatment, resulting in blurred ToF-SIMS images of both positive ions and negative ions (Fig. 2A). After ToF-SIMS imaging, the same cells were treated by air-plasma and the same spot was analyzed again by ToF-SIMS, revealing that air-plasma treatment reduced the background signals sufficiently that ToF-SIMS images of the cells became clear, as shown in Fig. 2B. Despite the improvement by air-plasma treatment for air-dried cells, only graphene-removed cells better maintained the morphology, as shown in Fig. 2C. Intriguingly, ToF-SIMS images better represented the whole cell morphology than optical images, in which we can only observe thick cell body parts rather than the thin leading edges. Consequently, our novel method enhances ToF-SIMS imaging so that spatial distribution of multiple lipids in well-preserved cellular membrane can be clearly observed. If secondary ion intensities are not high enough to make clear images, we can increase scan-number in ToF-SIMS imaging as much as signal-to-noise ratio is sufficiently high.

Plasma treatment is actually supposed to only clean hydrocarbon contaminations on the surface^22,23^ rather than removing graphene, and could also damage cellular membranes and modify surface properties, influencing SIMS analysis. For these reasons, how graphene can be removed and to what extent cells are effected by air-plasma will be discussed in the following section.

### Effect of air-plasma treatment on graphene-covered cells

**Fig. 3:**
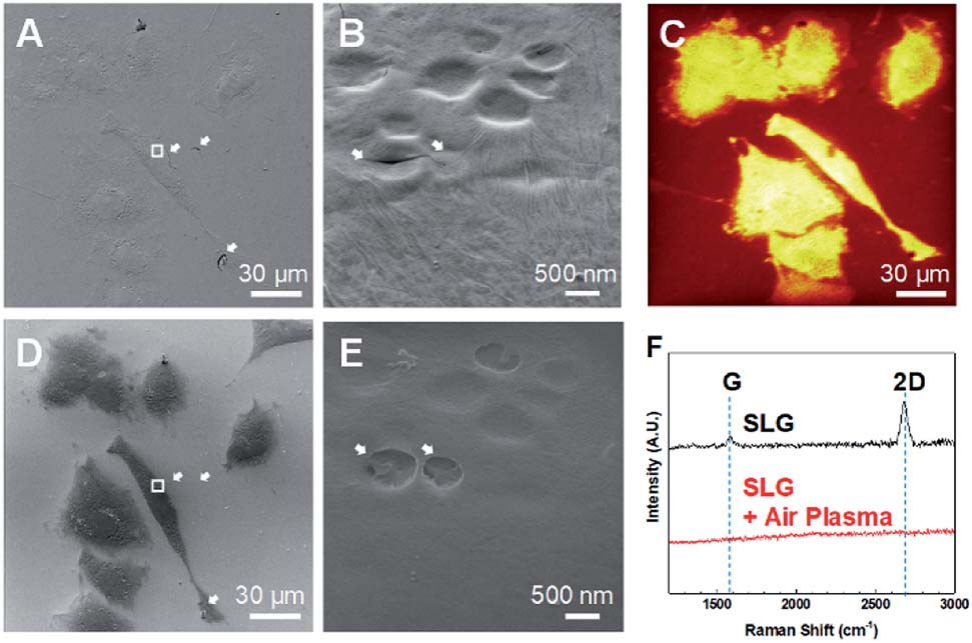
Graphene removal by air-plasma treatment. (A and B) HIM images for graphene-covered fixed A549 cells. White arrows in the HIM images indicate cracks and wrinkles in graphene. (C–E) ToF SIMS image of total positive ion (C) and HIM images (D and E) for graphene-removed A549 cells by 5 min air-plasma treatment. Cracks and wrinkles in graphene disappeared after graphene removed by air-plasma, as indicated with white arrows in the HIM images. The enlarged HIM images (B and E) were acquired from the area indicated with a white rectangle in low-magnified HIM images (A and D). (F) Raman spectroscopy of graphene-covered samples before and after 5 min air-plasma treatment. Scale bar, 30 μm (A, C and D), 500 nm (B and E).

Fixed graphene-covered A549 cells were examined using HIM (Fig. 3A and B). It has been reported that graphene enables electron microscopy imaging of intact nanoscale surface features of biological samples without any metal coating.^16,24^ Graphene also works for HIM imaging not only to image biological samples without distortion due to charging effects,^19^ but also to observe graphene surface itself with high contrast.^21^ We confirmed the high quality of graphene transfer on the fixed A549 cells with some trivial cracks, as shown in the HIM image (Fig. 3A). Graphene of a one-atom-thick layer follows precise surface features of samples that have relatively low aspect-ratio structures, and has nanoscale water droplets randomly trapped inside as well as irregular wrinkles, as shown in the enlarged HIM image (Fig. 3B). The water droplets between graphene and cellular membrane surface indicate that cells are kept wet in solution beneath graphene a while,^16,17^ thereby maintaining cellular volumes even under vacuum environment during HIM measurement.

We measured the same spot using HIM (Fig. 3D) after 5 min air-plasma treatment as the HIM (Fig. 3A) and ToF-SIMS analyzed region (Fig. 3C). Fig. 3D shows that graphene was completely removed from cell membranes, which was also confirmed by Raman spectra^25,26^ as shown in Fig. 3F. Bare biological samples without conductive material coating can also be imaged using HIM by compensating the charging effects by electron flood gun. With graphene removal, cracks and wrinkles in the graphene-cover disappeared, and the abundance of secondary electrons emitted from cells was reduced, making the cells well-distinguished from the surrounding glass substrate (Fig. 3D). Bare surface of cell membrane after 5 min air-plasma treatment retained the same surface features as before graphene removal and seemed to have no serious damages, as shown in Fig. 3B and E.

However, we cannot exclude the possibility that energetic air-plasma for cleaning hydrocarbon contaminations could damage biological samples, thereby affecting ToF-SIMS analysis. Thus, we studied the degrees of damage to cells by air-plasma depending on treatment time by observing the thin leading edge of cells using HIM. It turned out that relatively longer treatment, for 3 and 5 min, etched away some parts of the thinnest leading edge (Fig. S2A and B†), whereas 1 min treatment was still capable of completely removing graphene and did not cause noticeable damages as shown in HIM images (Fig. S2C†).

It is not likely that graphene was etched away by chemical reaction with plasma, because plasma treatment set for general cleaning process is not suitable to elicit reactive etching of graphene, and can only remove hydrocarbon impurities on graphene surface.^22,23^ It has been reported that graphene can be peeled off out of SiO_2_ substrate due to gas evolution and expansion between sample surface and graphene by hydrogen plasma,^27^ and that suspended graphene membranes inflate and bulge up due to pressure difference induced by energetic electron beam irradiation during scanning electron microscopy imaging.^28^ Based on the aforementioned reports, we speculate that a thin liquid layer or gas molecules stuck between graphene and sample surface could be activated by absorbing energy from energetic air-plasma, leading to gas expansion, so that graphene virtually placed on wet cells instead of being strongly adhered on them could be physically peeled off by a pressure difference during air-plasma treatment.

### Effect of air-plasma treatment on ToF-SIMS imaging of graphene-removed cells

**Fig. 4:**
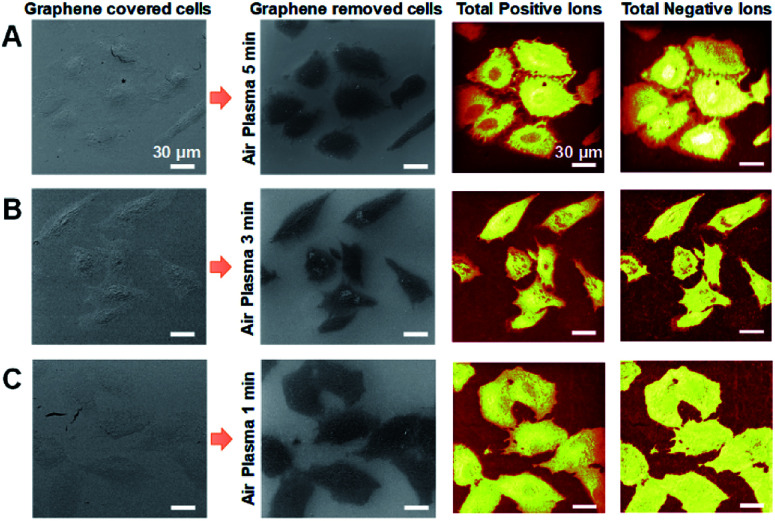
(A–C) HIM images and ToF-SIMS images of graphene-removed cells by air-plasma treatment for 5 min (A), 3 min (B) and 1 min (C). Scale bar, 30 μm (A–C).

**Fig. 5:**
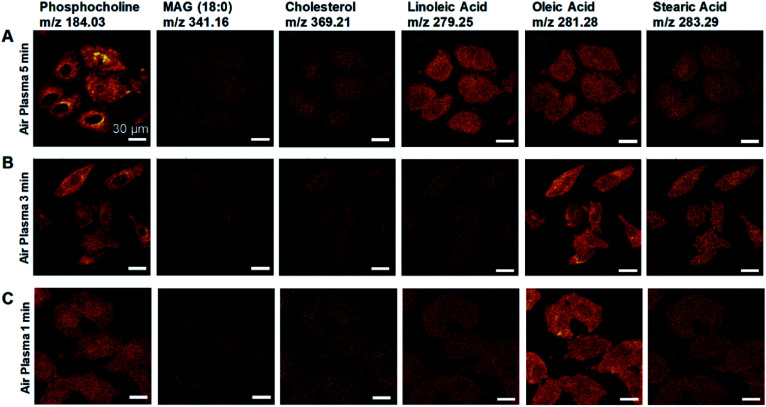
Effects of air-plasma treatment on ToF-SIMS imaging for graphene-removed cells. (A–C) ToF-SIMS images of (left to right) phosphocholine, monoacylglycerol 18 : 0, cholesterol, linoleic acid, oleic acid and stearic acid for graphene removed A549 cells by air plasma treatment for 5 min (A), 3 min (B) and 1 min (C). Scale bar, 30 μm (A–C).

Air-plasma treatment removed a graphene-cover from a cellular sample and then cleaned hydrocarbon contaminations on surface, yet when the treatment lasts longer, it can also erode the sample surface. To understand how much ToF-SIMS analysis is affected by air-plasma treatment, we prepared three graphene-removed A549 cells treated by air-plasma for 1, 3, and 5 min, respectively. ToF-SIMS images of total positive ions and negative ions, and HIM images before and after air-plasma treatment for each sample, were displayed in Fig. 4. HIM images show that graphene was successfully transferred onto cells and completely removed from all of the samples, and ToF-SIMS images of high resolution and high contrast clearly represent the whole cell morphology, which corresponds roughly to ones as in HIM images. We also acquired ToF-SIMS images of multiple lipids in parallel, such as phosphocholine, monoacylglycerols, cholesterol, and several fatty acids, parts of which are displayed in Fig. 5. All of the ToF-SIMS images of lipids did not show any visible differences between samples except for phosphocholine ion. As air-plasma treatment lasts longer (*i.e.*, over 1 min), phosphocholine seems to get reduced on the center region of cell membrane near the nucleus (Fig. 5A and B), whereas the cell body region is slightly more enriched with phosphocholine than the outside area of the whole cell for 1 min-treated cells (Fig. 5C). Reduced phosphocholine for 5 min-treated cells is likely due to damage on cell membrane by long air-plasma treatment, which eventually distorts ToF-SIMS analysis.

## Conclusions

In this paper, we studied cellular sample preparation methods based on air-plasma treatment to obtain high spatial resolution ToF-SIMS imaging of cells. Although two steps, graphene covering and air-plasma treatment, were added to the normal air-drying sample preparation, the proposed sample preparation process was not difficult and certainly led to clear ion imaging results. The air-plasma treatment in cellular specimen preparation, not only effectively removed background spectra from the surface of the sample and substrate, but also peeled off the graphene layer from the cellular specimen, resulting in improved ToF-SIMS images of the cells. The proposed cellular specimen preparing protocol suitable for a high-vacuum environmental equipment can be applied to a variety of cell and tissue analyses, such as investigation of the constituents of a bio-substance, observation of the microstructures and morphology, diagnostics of presence or absence of disease, and detection of drug or pesticide.

## Conflicts of interest

There are no conflicts to declare.

## Supplementary Material

RA-009-C9RA05205D-s001
