# Supplementary material for: Preparation of cellular samples using graphene cover and air-plasma treatment for time-of-flight secondary ion mass spectrometry imaging
Source: RSC Adv. 2019 Sep 9;9(49):28432–8. doi: 10.1039/c9ra05205d (PMC9071169; doi:10.1039/c9ra05205d)
Supplement: RA-009-C9RA05205D-s001 [file RA-009-C9RA05205D-s001.pdf]

Electronic Supplementary Material (ESI) for RSC Advances.
This journal is © The Royal Society of Chemistry 2019

Supplementary Online Information for

Preparation of cellular samples using graphene cover and air-plasma treatment for time-of-flight secondary ion mass spectrometry imaging

Heejin Lim, Sun Young Lee, Dae Won Moon*, and Jae Young Kim*

Supplementary Figures

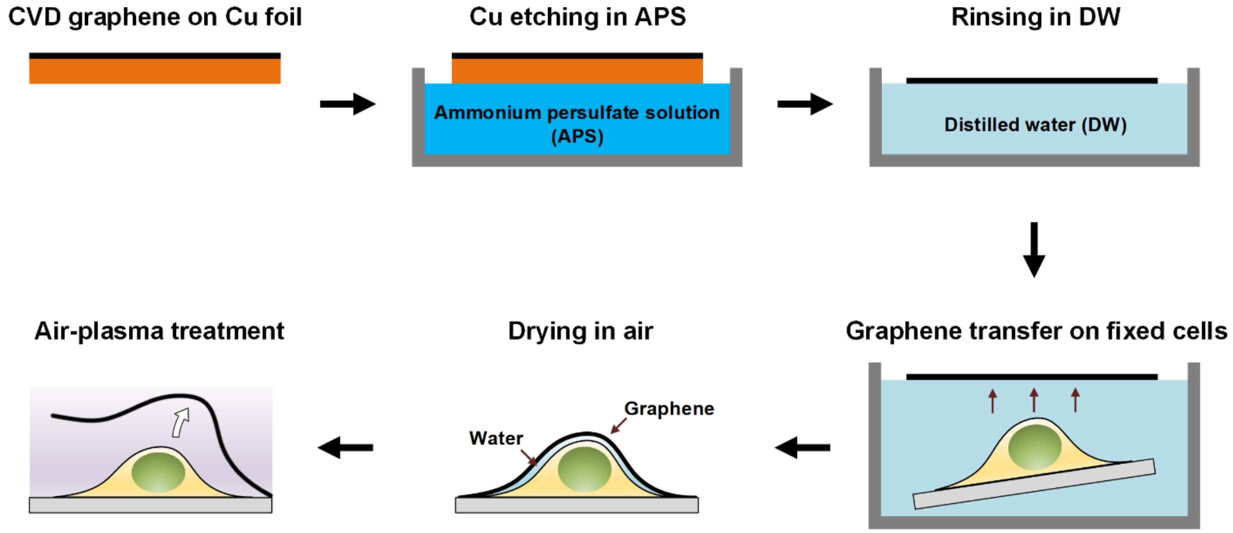

Figure S1. Entire process for graphene transfer on cells and air-plasma treatment.

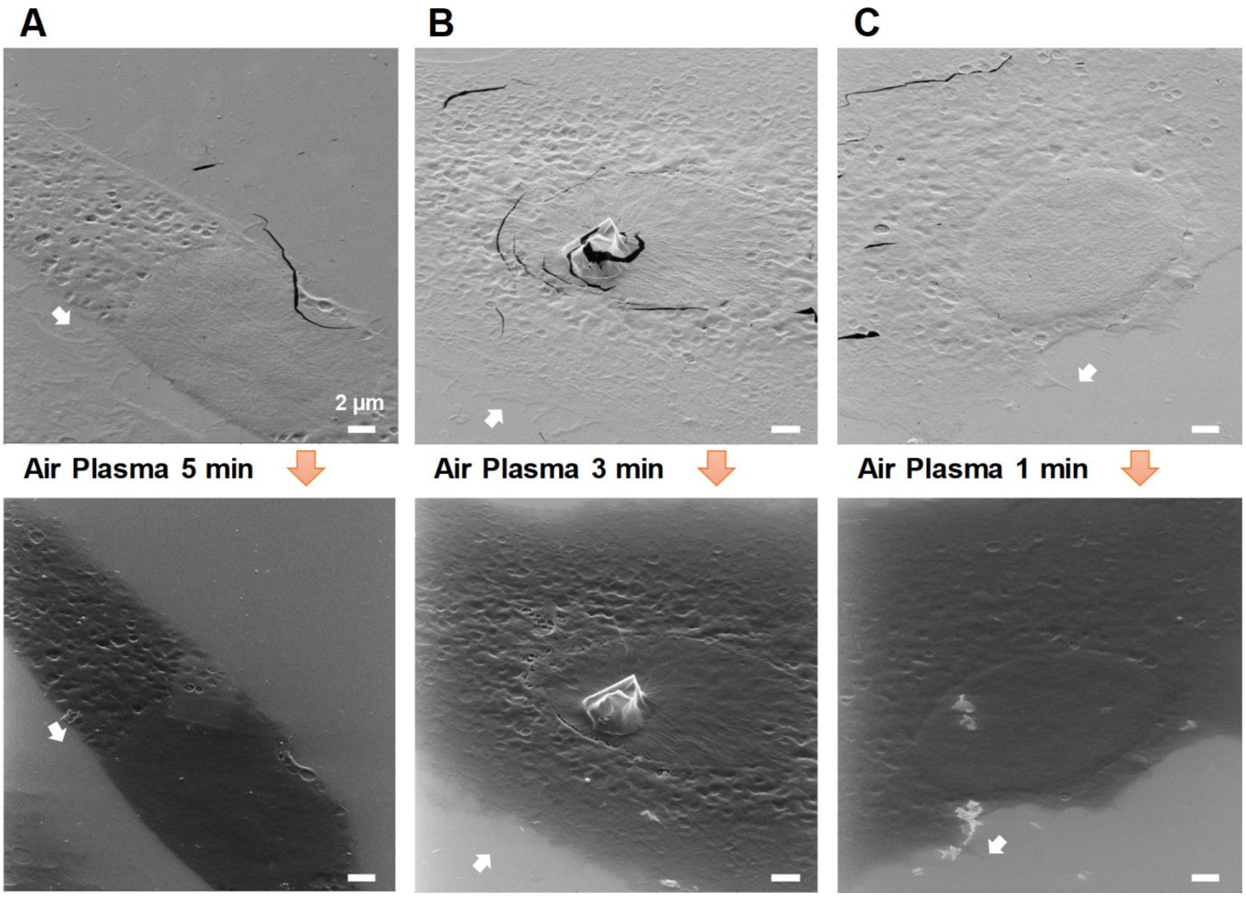

Figure S2. Effects of air-plasma treatment on graphene-covered cells. A-C, HIM images of graphene-covered fixed A549 cells before (upper) and after air-plasma treatment (lower) for 5 min (A), 3 min (B), and 1 min (C). Treatment of air-plasma over 1 min can not only remove graphene, but also damage cellular membrane. White arrows (A, B) show some part of cellular leading edges were etched away by air-plasma. In contrast, 1-min air-plasma treatment is sufficient to completely remove graphene without noticeable damage. Scale bar, 2 μm (A-C).
